# MADIBA: A web server toolkit for biological interpretation of Plasmodium and plant gene clusters

**DOI:** 10.1186/1471-2164-9-105

**Published:** 2008-02-28

**Authors:** Philip J Law, Clotilde Claudel-Renard, Fourie Joubert, Abraham I Louw, Dave K Berger

**Affiliations:** 1 Bioinformatics and Computational Biology Unit, African Centre for Gene Technologies (ACGT), Department of Biochemistry, Faculty of Natural and Agricultural Sciences, University of Pretoria, Pretoria, 0002, South Africa; 2 Department of Plant Science, Forestry and Agricultural Biotechnology Institute (FABI), University of Pretoria, Pretoria, 0002, South Africa; 3 Laboratoire de Biologie Cellulaire et Moléculaire, Biogemma, Domaine de Sandreau, 31700, Mondonville, France

## Abstract

**Background:**

Microarray technology makes it possible to identify changes in gene expression of an organism, under various conditions. Data mining is thus essential for deducing significant biological information such as the identification of new biological mechanisms or putative drug targets. While many algorithms and software have been developed for analysing gene expression, the extraction of relevant information from experimental data is still a substantial challenge, requiring significant time and skill.

**Description:**

MADIBA (MicroArray Data Interface for Biological Annotation) facilitates the assignment of biological meaning to gene expression clusters by automating the post-processing stage. A relational database has been designed to store the data from gene to pathway for *Plasmodium*, rice and *Arabidopsis*. Tools within the web interface allow rapid analyses for the identification of the Gene Ontology terms relevant to each cluster; visualising the metabolic pathways where the genes are implicated, their genomic localisations, putative common transcriptional regulatory elements in the upstream sequences, and an analysis specific to the organism being studied.

**Conclusion:**

MADIBA is an integrated, online tool that will assist researchers in interpreting their results and understand the meaning of the co-expression of a cluster of genes. Functionality of MADIBA was validated by analysing a number of gene clusters from several published experiments – expression profiling of the *Plasmodium *life cycle, and salt stress treatments of *Arabidopsis *and rice. In most of the cases, the same conclusions found by the authors were quickly and easily obtained after analysing the gene clusters with MADIBA.

## Background

A greater understanding of the biological mechanisms within organisms becomes possible with the availability of complete genome data, in combination with high-throughput screening methodologies such as microarrays. In addition, numerous databases provide annotation at different biological levels. These include databases on the annotation of genes according to the Gene Ontology (GO) nomenclature [1], metabolic pathways as in KEGG [2], or Transcription Factor Binding Sites (TFBS) in TRANSFAC [3] to annotate promoters.

Generally, gene expression data are normalised, filtered and finally genes with similar expression profiles are clustered into groups. The biological hypothesis behind this is that similarly expressed genes have a common biological characteristic, for example participation in the same biological process, or regulation by a common transcription factor.

Several currently available tools provide an interpretation of gene clusters but are often specialised in their analyses. For example, FatiGO [4], GeneLynx [5] and Gostat [6] are powerful tools for GO term identification; GoMiner [7], MAPPFinder [8] and DAVID [9] propose GO and metabolic pathway interpretation; MiCoViTo [10] proposes metabolic pathways and incorporates transcription regulation visualisation; metaSHARK [11] predicts enzyme-coding genes from unannotated genome data and places them on generic metabolic pathways; and WebGestalt [12] uses data obtained from different public resources and offers an integrated platform to perform various analyses such as a GO analysis, metabolic pathways and chromosomal distributions.

To facilitate the analysis of gene expression experiments, we have developed MADIBA (MicroArray Data Interface for Biological Annotation), a web based interface with a relational database that currently provides five analytical modules to assist researchers in the identification of possible reasons for the common expression of a cluster of genes. These modules are: (1) a search of over-represented GO terms in the cluster; (2) mapping of the cluster's gene products onto metabolic pathways using the KEGG representation; (3) visualisation of the chromosomal localisation; (4) a search of over-represented motifs in the upstream sequences of the genes and (5) an organism specific analysis.

MADIBA has currently been implemented for *Plasmodium falciparum*, *Oryza sativa *(rice) and *Arabidopsis thaliana*. Malaria is a devastating disease, particularly in Africa, so understanding how its causative agent, *Plasmodium*, functions is essential. Rice and *Arabidopsis *are model species for monocotyledonous and dicotyledonous plants respectively [13], and plant analyses are useful particularly for gaining insights into improving crops in both developed and developing countries, for example orphan crops such as cassava, cowpea and pearl millet, which are important for food security in Africa. In addition, *Plasmodium *is related to plants as the apicoplast (apicomplexan plastid) is reminiscent of the chloroplast [14,15].

## Construction and content

### User interface

MADIBA is accessible through a simple and user friendly web interface [see Additional file 1]. Once a set of sequences or gene identifiers has been submitted, the user is provided with links to the five analysis modules and the output module. Each analysis module is independent of the others and is accessed individually. In addition, the genes that are to be used in subsequent analyses are listed.

### Data submission

A cluster of genes is submitted to MADIBA, either by uploading a file, or directly pasting a set of nucleotide sequences, in FASTA format. Alternatively, a list of gene identifiers can be submitted. The gene clusters are obtained from any clustering algorithm, such as hierarchical or *k*-means, since MADIBA does not perform any clustering.

For *Plasmodium *and *Arabidopsis *sequences, a BLASTN search is performed, and a BLASTX search of the rice sequences is performed to allow the possibility of entering gene clusters from the *indica*, as well as the *japonica*, subspecies. In addition, this will potentially allow orthologous gene clusters from other cereals to be analysed, such as pearl millet. Users select which of the BLAST hits they wish to continue the analyses with, and this list of genes is stored.

**Figure 1:**
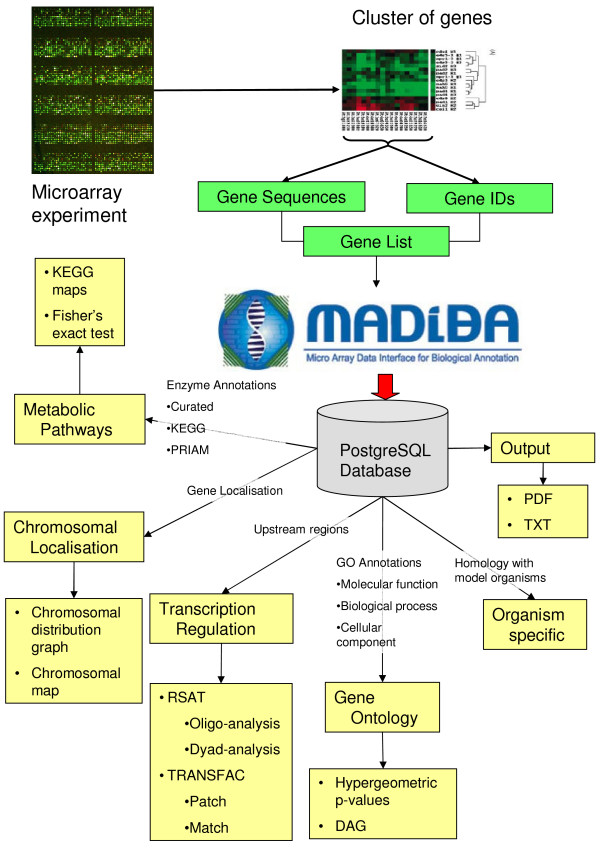
**A schematic representation of the flow of data through MADIBA**. After a microarray experiment, data are normalised and then clustered, since it is hypothesised that the genes in a cluster have common biological implications. A cluster of genes is submitted to MADIBA, either as nucleotide sequences, or gene identifiers. This list of genes can then be subjected to five analysis modules – Gene Ontology Analysis, Metabolic Pathways Analysis, Transcription Regulation Analysis, Chromosomal Localisation Analysis and an Organism Specific Analysis. Also shown are the data that are required by each of the analysis modules. The results from the analyses can be exported as a PDF file, or as plain text.

The gene list is stored for one week on the server, and a unique identifier is provided to allow users to later access and retrieve their data. As each analysis module is accessed, the gene list is used to retrieve the necessary information required by that module from the database. Figure 1 illustrates the architecture and basic data flow of an analysis in MADIBA as described in the next section.

### MADIBA modules to analyse gene clusters

#### Gene Ontology module

This analysis module extracts the GO annotations according to the molecular function, biological process and cellular component ontologies. For a given ontology, a Directed Acyclic Graph (DAG) is drawn in a single view to show the genes from the cluster, and the GO terms that they are annotated to. Each GO term that is found in the cluster is drawn to show its position in the GO hierarchy, in a manner similar to AmiGO's graphical representation [16] and GO::Termfinder [17]. The user is able to select which genes should be visualised, to prevent overly complex graphs.

An adjusted hypergeometric *p*-value for each annotation is also calculated to evaluate the significance of the annotation. The adjustment methods used are a False Discovery Rate (FDR) adjustment [18], and a Holm adjustment [19], which works under the same assumptions as a Bonferroni correction, but is statistically more powerful [20]. Nodes on the DAG are coloured according to its FDR corrected *p*-value, where red indicates *p*-value ≤ 10^-10^; orange, 10^-10 ^<*p*-value ≤ 10^-8^; yellow, 10^-8 ^<*p*-value ≤ 10^-6^; green, 10^-6 ^<*p*-value ≤ 10^-4^; blue, 10^-4 ^<*p*-value ≤ 10^-2^; purple, *p*-value > 10^-2^. Other statistical tests will be implemented in the future to provide greater flexibility to the user in calculating the enrichment of the GO terms. A tab delimited text file with each gene and the GO terms associated is provided should users wish to perform their own analyses.

#### Metabolic Pathways module

When this module is accessed, a list of all the KEGG metabolic pathways is presented, along with an indication of how many enzymes encoded by genes from the input cluster were found in each pathway. Each pathway in the list is linked to its diagram where the protein products of the genes in the cluster are highlighted. This module compares the enzymatic annotation from three different annotation sources, namely the curated annotation from the original data source (PlasmoDB, TIGR or TAIR); the semi-automatic KEGG annotation and the automatic PRIAM annotation [21]. The use of these diverse and independent annotations increases the robustness of the analysis. Different colours are used to indicate the agreement between the three annotation methods, where yellow indicates that the enzyme was annotated by all three annotation sources; red, by any two annotations; blue, by KEGG only; purple, by PRIAM only; and green, by the original annotators only. In addition, any enzyme found in the genome annotation, but not in the cluster is coloured grey. All elements on the pathways are linked to the KEGG website to provide more information on the particular enzyme or compound. Clicking on an enzyme that is present in the cluster also provides information as to which annotations were used to describe it, and which genes from the cluster encode it.

A *p*-value is calculated for each pathway using Fisher's exact test [22] to indicate the significance of the pathways, using a 2 × 2 contingency table where the rows indicate pathway membership and the columns indicate cluster membership [23].

#### Chromosomal Localisation

This module permits the identification of co-expressed genes on the same chromosomal region. It provides a bar chart showing the distribution of the genes in the cluster across the chromosomes, that is, the number of genes on each chromosome. In addition, a schematic visualisation of the genes along the chromosomes is provided, where each chromosome is drawn as a horizontal bar, and each gene is represented by a vertical blue line. The size of the bar is relative to the size of the chromosomes, and the genes are drawn in a location with respect to its actual position on the chromosome. Localisation data was obtained from the original annotation source (PlasmoDB, TIGR or TAIR). A mouse-over effect was included to this diagram to allow easier identification of a gene at a particular position.

#### Transcription Regulation module

This module presents an approach of motif identification without any prior knowledge, by automatically detecting potential Transcription Factor Binding Sites (TFBS) in the promoter sequences of co-regulated genes, using Regulatory Sequence Analysis Tools (RSAT) [24], specifically using the oligo-analysis and dyad-analysis programs. Oligo-analysis calculates the occurrence of words (oligonucleotides) in a set of sequences, and determines which are overrepresented based on a background model. Dyad-analysis detects overrepresented spaced dyads (oligonucleotide pairs which are separated by a variable spacer region) in a set of sequences. For both analyses, the five most significant motifs are reported, with the option to view all results.

The upstream regions are also searched for known TFBS in the TRANSFAC database (Professional version 11.1) using the built-in Patch and Match programs [25]. Patch uses predefined binding site entries and performs a pattern-based binding site search, while Match uses positional weight matrices derived from alignments of binding sites (i.e. matrix-based search). The ten most common motifs found by each tool are presented. For each identified binding site, a link is provided to additional information in TRANSFAC about the binding factor.

#### Organism Specific Characteristics module

In the *Plasmodium *cluster analyses, a component of the aim for this module is to identify putative new drug targets. Thus, a list of the genes without human homologues, with their respective annotations is generated. Also, if any genes similar to the apicoplast are present, its closest homologue to *Arabidopsis *is identified. Due to its vegetal nature, the apicoplast may provide a target for herbicide-like drugs which will not affect the human host [14,15].

For the plant analyses, the closest *Arabidopsis *orthologue of each rice gene, and *vice versa*, is given, in an effort to identify similar genes. This was accomplished by implementing a reciprocal BLASTP search, with a stringent e-value cut-off of 10^-15 ^to identify highly probable orthologous proteins. In addition, a list of all similar genes, based on sequence similarity is returned, representing the paralogues, or protein 'families', for each gene. These results are determined by performing a self BLAST, and the user is able to determine the most relevant results by choosing their own e-value cut-off, minimum percent coverage (how much of the query matched the subject) and minimum percent identity (how much of the match corresponded).

### Output

Results from MADIBA can be exported in either plain text or PDF formats. The user selects the required set of results which is then generated for immediate download.

### Implementation

The MADIBA interface was written in PHP and Python-CGI, and the graphical outputs are dynamically generated using the GD library [26]. A central PostgreSQL database is used to store the downloaded and pre-calculated data.

The downloaded data currently consists of data from the PlasmoDB [27] database for *Plasmodium falciparum *(release 5.2), TIGR [28] for rice (*Oryza sativa *ssp *japonica cv Nipponbare*) (Osa1 database release 5) and TAIR [29] for *Arabidopsis thaliana *data (TAIR7). We store the gene name, functional annotation, GO identifiers, chromosomal localisations, and the enzymatic annotations (EC identifiers) from the above data sources, as well as the EC numbers proposed by the KEGG Orthology results.

Data pre-calculated by programs before being stored in the database, include putative metabolic enzyme predictions using PRIAM [21]. These predictions are calculated based on enzyme profiles from position specific weight matrices. Also stored are the 1500 nucleotides upstream of the start codon (ATG) based on previous experimental research on *Plasmodium *promoters [30-33], and 1000 nucleotides upstream of the rice and *Arabidopsis *genes, as made available by TIGR and TAIR respectively. We also identify putative orthologues between *Plasmodium falciparum *and human proteins, and rice and *Arabidopsis *proteins, by performing a reciprocal BLASTP search, with an e-value cut-off of 10^-15^. In addition, for rice and *Arabidopsis*, a BLASTP of all expressed proteins was performed against the proteome of the organism in question (a self BLAST), in order to determine paralogues, or protein 'families', for each gene. The BLAST results with an e-value less than 10^-3 ^were stored in the database. Sequence information was obtained from the original data source, and BLASTs were performed using a local version of NCBI-BLAST.

The DAGs from the GO analysis are drawn using the *dot *program in Graphviz [34], and visualised using the ZGRViewer applet [35]. All statistical calculations (hypergeometric and Fisher's tests, and multiple hypothesis corrections) were computed by the R statistics package [36], which was accessed using the RPy package [37], a Python interface to R. The KEGG maps are coloured using the Python Imaging Library [38], using the co-ordinates provided by KEGG. The chromosomal distribution bar chart and chromosomal schematic are drawn using the PHP GD library. Both RSAT and TRANSFAC are accessed using system calls. The PDF output files are created using ReportLab [39].

## Utility

### Plasmodium data analysis

The results of an oligonucleotide array profiling the expression of human and mosquito stages of the malaria parasite's life cycle [40] were chosen to demonstrate the functionalities of MADIBA. After a robust *k*-means clustering algorithm, 15 clusters were proposed, and these clusters were analysed with MADIBA.

**Figure 2:**
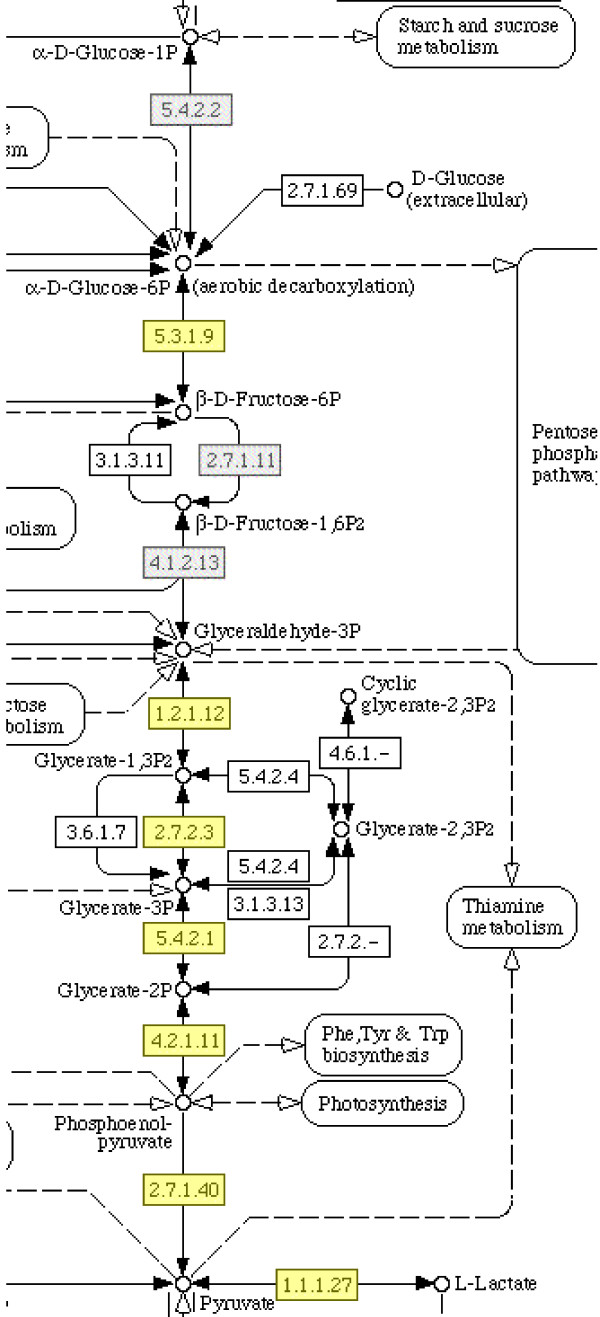
**Results from the Metabolic Pathways module**. Analysis of cluster 6 of the *Plasmodium *data [40] revealed that it was noticeably involved in glycolysis. In the KEGG map for glycolysis, it could be seen that almost all of the enzymes involved are present in the cluster. Additionally, all of the enzymes were annotated by all the three annotation sources – the curated annotation from the original data source (PlasmoDB); the semi-automatic KEGG annotation and the automatic PRIAM annotation, as indicated by the yellow boxes.

**Figure 3:**
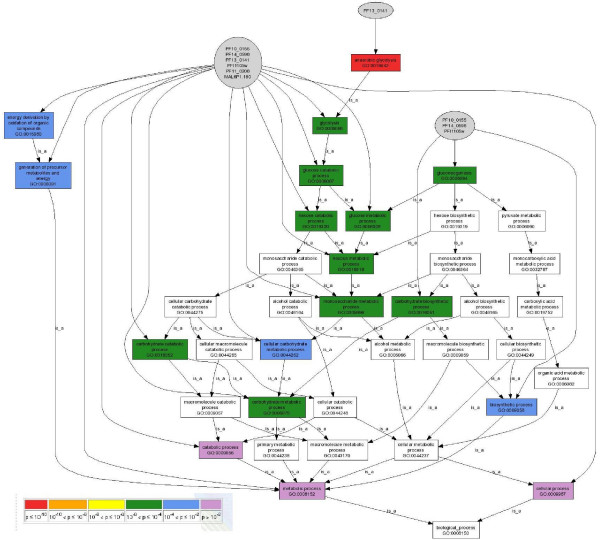
**Results from the Gene Ontology module**. An analysis of the biological process ontology of the cluster 6 of the *Plasmodium *data [40] revealed that anaerobic glycolysis was the most significant term. The DAG was reduced to show only the terms that are most relevant to glucose metabolism. The grey ellipses contain the genes that are annotated to the connected GO term and the colour of the GO terms indicates different levels of significance, as indicated by the legend.

After applying MADIBA, an improvement in the number of annotated genes is apparent compared with the original results. The mean fraction of known genes by cluster was 37.5% compared with 41% when using MADIBA. The Gene Ontology module automatically allocated annotations to the gene clusters with terms including immune evasion, in cluster 1, and cell invasion in cluster 15. In addition, the genes in cluster 2 were correctly identified as involved in gametogenesis and having over-represented protein kinase cascade activity. The metabolic pathways module successfully showed that six of the nine enzymes in the glycolysis pathway were found in cluster 6, with a *p*-value of 0.04, as calculated by using Fisher's exact test (Figure 2). This result is further supported by the indication that all the enzymes in the pathway were identified by all three annotation sources, as indicated by the yellow boxes, and by using the GO analysis, it was shown that the anaerobic glycolysis term had a highly significant *p*-value (Figure 3 and Table 1). Using the module specific for *Plasmodium *characteristics allowed the identification of genes in cluster 3 as interesting drug or vaccine targets, such as PF10_0303, an ookinete surface antigen.

**Table 1:** A portion of the table of *p*-values that accompany the DAG (Figure 3) from the analysis of the biological process ontology of cluster 6 of the *Plasmodium *data [40], showing the *p*-value calculated from the hypergeometric distribution, along with the Holm and FDR multiple hypothesis corrections.

Term	Definition	Corrected p-value (FDR)	Corrected p-value (Holm)	Uncorrected p-value
GO:0019642	anaerobic glycolysis	0	0	0
GO:0006096	Glycolysis	3.6675E-06	0.00010971	7.2180E-07
GO:0005996	monosaccharide metabolic process	5.3464E-06	0.00016747	1.1091E-06
GO:0019318	hexose metabolic process	5.3464E-06	0.00016747	1.1091E-06
GO:0006094	gluconeogenesis	6.7072E-06	0.00021795	1.4627E-06
GO:0016051	carbohydrate biosynthetic process	6.7072E-06	0.00021795	1.4627E-06

Young *et al*. [41] performed an analysis on the transcriptome of *Plasmodium *in an attempt to identify hypothetical genes that are likely to be involved in the sexual development of the parasite. Using an algorithm called ontology-based pattern identification (OPI) on the data, a set of 246 genes were grouped as interesting. Applying RSAT from the Transcriptional Regulation module on this data set identified several overrepresented motifs in the upstream regions of the genes. In particular, oligo-analysis identified the motif GATGAA, which had an expected occurrence of 96.6 based on the background model, but occurred 228 times (E-value for occurrence 10^-26^). Similarly with dyad analysis, the motif ATCN{7}TCA was found to occur 154 times, as opposed to its expected occurrence of 41.3 (E-value for occurrence 3.5 × 10^-32^).

### *Arabidopsis *data analysis

**Figure 4:**
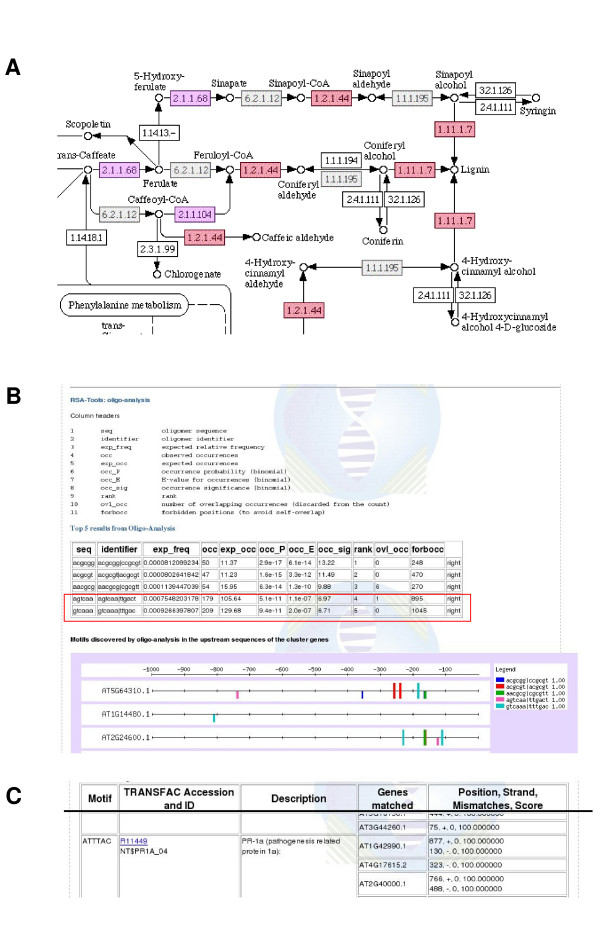
**Results from the *Arabidopsis *data**. (A) Analysis of cluster 0 from the *Arabidopsis *salt stress experiment [42] with the Metabolic Pathways module revealed that the cluster contained genes involved in lignin biosynthesis. The red colour indicates that the annotations were found by two annotation methods (PRIAM and KEGG in this case), and the purple indicates the enzyme was annotated by PRIAM only. (B) After analysing cluster 8 of the *Arabidopsis *data [42] with the Transcription Regulation module, it was possible to identify putative transcription factor binding sites. The output of the oligo-analysis tool of RSAT is shown, indicating two motifs on the reverse complement that were identified as similar to the WRKY binding site ((C/T)TGAC(T/C)) (highlighted in the red box). Cluster 8 is known to contain several WRKY transcription factors and several disease-resistance genes. (C) Output from the Patch program of the TRANSFAC sub-module. Shown is the PR-1a (a pathogenesis related protein) promoter binding site that was identified. The table headers are provided for convenience.

MADIBA was used to analyse data from a study of the response of *Arabidopsis *to salt stress [42]. The genes were clustered using a "fuzzy k-means clustering" into 10 major clusters. After analysing individual clusters with MADIBA, it was found that the analyses supported the authors' conclusions. An example is cluster 0 that had genes responding to osmotic stress in leaves and salt stress in roots, and meta-analysis with other array data by the authors led them to conclude that it contained many biotic stress response genes. Analysis with the Metabolic Pathways module of MADIBA showed over-representation of several enzymes involved with lignin biosynthesis in cluster 0 (*p *= 0.001) (Figure 4a), which is indicative of a defence response. The Gene Ontology analysis showed enrichment of terms in dihydrocamalexic acid decarboxylase activity, an enzyme responsible for the production of camalexin, a phytoalexin in *Arabidopsis *produced in response to pathogen infection [43]. Other enriched terms included chitinase activity, ion channel activity, and terms involved in calcium ion activity including ion binding terms and calmodulin binding. The calcium ion responsive terms are most likely related to the effect of salinity on the plant. In the molecular process ontology, terms included regulation of cellular defence responses, hypersensitive response, as well as several biotic stress indicators including responses to ethylene stimulus, jasmonic acid stimulus, salicylic stimulus and abscisic acid stimulus. These hormones are known to be involved in plant defences as well as playing roles in salt-stress signalling, again suggesting cross-talk between the various signalling responses. Cluster 8 was annotated as immediate response genes, and contained members of the WRKY transcription factor family and disease-resistance protein genes. Analysis by the oligo-analysis program of RSAT showed that on the reverse complement, the TTGACT and TTTGAC motifs were overrepresented in the cluster (E-values 1.1 × 10^-7 ^and 2 × 10^-7 ^respectively), which is similar to the WRKY binding site ((C/T)TGAC(T/C) [44]) (Figure 4b). In addition, analysis by the TRANSFAC subsection of the Transcription Regulation module showed that a large proportion of the genes (110 out of a total of 142 genes in the cluster) contained a motif (ATTTAC) that is functionally important in the promoter of PR-1a, a well characterised pathogenesis related protein [45] (Figure 4c).

### Rice data analysis

For a rice analysis, the results from a cDNA microarray of cultured rice cells in response to flagellin [46] were used. Using *k*-means clustering, 9 clusters were distinguished. The Gene Ontology analysis detected several genes involved in wounding and defence responses. Although the experiment was conducted using the *indica *subspecies of rice, the results still show an improvement in the annotation and illustrates the ability of the BLASTX search to find orthologous genes.

Additionally, a set of stress-induced rice genes from a transcriptome study on cold, drought, salinity and abscisic acid treatments were tested [47]. While the authors did not perform a statistical clustering, several sets of genes were identified as stress induced and grouped together. After analysing the complete gene set of 73 stress-induced genes with MADIBA, the Gene Ontology analysis identified terms such as responses to stress, cold acclimation, iron ion transport and water deprivation, as being significant, confirming the gene set to be stress responsive. Interestingly, several genes in the set were also annotated as responses to jasmonic- and salicylic-acid, which are usually considered to be signalling molecules in a plant's defence response. This suggests that there is some cross-talk between biotic and abiotic stress responses.

## Discussion

While other tools similar to MADIBA, such as WebGestalt, FatiGO and GoMiner exist, MADIBA differs in that it has a wider range of analyses which can be performed in an integrated fashion, for example, it performs a GO analysis as well as a Transcription Regulation analysis. Of the previously mentioned tools, MADIBA is most similar to WebGestalt [12], which also obtains information from different data sources and provides an integrated set of analysis tools to assist researchers in mining this gene set. WebGestalt, however, does not provide information on transcription regulation, and currently only works for human and mouse data. MADIBA is unique in the organisms it is able to analyse – *Plasmodium falciparum*, *Oryza sativa *(rice) and *Arabidopsis thaliana*. ClutrFree [48] is a desktop-based tool that uses a different approach to facilitate interpretation of microarray data. It is a flexible and generic platform that allows the user to compare different annotation and analysis approaches to a microarray data set. Pattern recognition allows visualisation of the relationships in a directed graph (tree) that assists the user in deriving biological conclusions.

Due to its modular nature, any new analysis can easily be added to MADIBA at a later stage. In addition, the current analysis modules are continually being improved to assist the user in identifying the reasons for the co-expression of a set of genes. Since it is a web application, this makes MADIBA platform-independent and can be accessed from anywhere in the world. Furthermore, the database can be updated, so that the latest information is available to the user. MADIBA is highly dependent on the quality of the genomes' annotations, so as the annotations are improved, so will the results returned by MADIBA.

### Future prospects

All the statistics performed on the data are analysed in terms of the entire genome. However, since whole genome microarray slides are not always used, a proposed improvement is to analyse the data in terms of only the genes that were on the slide.

MADIBA has been designed to be generic and easily expandable, so that any new organisms that are required by the community can readily be incorporated into the database, with only a fully annotated genome necessary. In addition, as the genome annotations are revised, it is important to update the data within MADIBA, and this will be done in a semi-automated manner using pre-built Python scripts.

With the increased number of statistical methods being adopted, we aim to widen the variety of statistical analyses available, such as by including GSEA [49] (through R) or rank tests, to provide a greater level of flexibility for the user.

In the Organism Specific module, orthology was inferred using reciprocal best BLAST hit. We recognise that this is not the most accurate or reliable method for determining orthology, so we are working to implement tools such as Ortholuge [50] and GreenPhyl [51], which take into account phylogenetic information in addition to sequence similarity.

## Conclusion

With the advent of whole genome microarray chips and an increasing number of organisms being sequenced, tools such as MADIBA will become even more significant in understanding the underlying biology. MADIBA provides access to several genomic data sources and analyses, allowing users to quickly annotate and visualise the results. Moreover, this tool can contribute information about *Plasmodium *as gene regulation in this parasite is poorly understood [52].

## Availability and requirements

MADIBA is freely available and can be accessed on the web using a JavaScript enabled browser at [53]. The home page provides links to an online tutorial, and demonstration gene cluster data and analysis results, including those discussed above.

## Authors' contributions

PJL was involved in expanding MADIBA to include the plant data, improving the metabolic pathways and GO modules, and prepared the manuscript. CCR initiated the project, was responsible for the *Plasmodium *data, and drafted the manuscript. FJ was responsible for bioinformatics and participated in its design. AIL conceived the study and participated in its design. DKB was involved in plant analyses, participated in its design and preparation of the manuscript. All authors have read and approved the manuscript.

## Supplementary Material

Additional file 1 MADIBA initial submission page. A screen shot of MADIBA after a set of sequences has been submitted. Block A illustrates the links to the five analysis modules and the output module, Block B shows the unique identifier that is provided to the user and section C lists the genes that are to be used in subsequent analyses. Click here for file
