# Supplementary figures and images for: MADIBA: A web server toolkit for biological interpretation of Plasmodium and plant gene clusters
Source: BMC Genomics. 2008 Feb 28;9:105. doi: 10.1186/1471-2164-9-105 (PMC2277412; doi:10.1186/1471-2164-9-105)

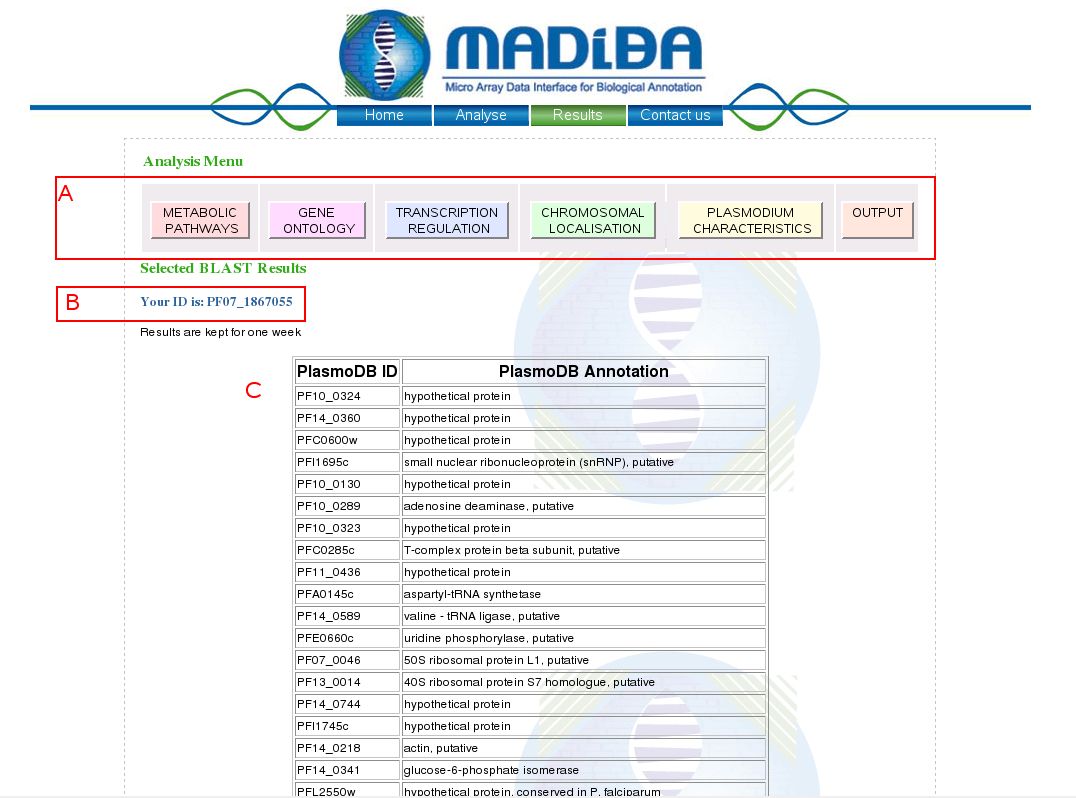

Supplement: Additional file 1 — MADIBA initial submission page. A screen shot of MADIBA after a set of sequences has been submitted. Block A illustrates the links to the five analysis modules and the output module, Block B shows the unique identifier that is provided to the user and section C lists the genes that are to be used in subsequent analyses. [file 1471-2164-9-105-S1.jpeg]
